# Combining host and rumen metagenome profiling for selection in sheep: prediction of methane, feed efficiency, production, and health traits

**DOI:** 10.1186/s12711-023-00822-1

**Published:** 2023-07-25

**Authors:** Melanie K. Hess, Larissa Zetouni, Andrew S. Hess, Juliana Budel, Ken G. Dodds, Hannah M. Henry, Rudiger Brauning, Alan F. McCulloch, Sharon M. Hickey, Patricia L. Johnson, Sara Elmes, Janine Wing, Brooke Bryson, Kevin Knowler, Dianne Hyndman, Hayley Baird, Kathryn M. McRae, Arjan Jonker, Peter H. Janssen, John C. McEwan, Suzanne J. Rowe

**Affiliations:** 1 University Invermay Agricultural Centre, AgResearch Ltd., Private Bag 50034, Mosgiel, 9053 New Zealand; 2 University of Nebraska-Lincoln, Institute of Agriculture and Natural Resources, 300 Agricultural Hall, University of Nebraska-Lincoln, Lincoln, NE 68583 USA; 3 Wageningen University & Research, P.O. Box 338, 6700 AH Wageningen, The Netherlands; 4 University of Nevada, Reno, Agriculture, Veterinary & Rangeland Sciences, 1664 N. Virginia St., Mail Stop 202, Reno, NV 89557 USA; 5 Graduate Program in Animal Science, Universidade Federal do Pará (UFPa), Castanhal, Brazil; 6 Ruakura Research Centre, AgResearch Ltd., Private Bag 3115, Hamilton, 3240 New Zealand; 7 Deer Industry New Zealand, PO Box 10702, Wellington, 6140 New Zealand; 8 Pāmu, Landcorp Farming Ltd, PO Box 5349, Wellington, 6011 New Zealand; 9 Woodlands Research Farm, AgResearch Ltd., 204 Woodlands-Morton Mains Road, Woodlands, 9871 New Zealand; 10 Grasslands Research Centre, AgResearch Ltd., Private Bag 11008, Palmerston North, 4410 New Zealand

## Abstract

**Background:**

Rumen microbes break down complex dietary carbohydrates into energy sources for the host and are increasingly shown to be a key aspect of animal performance. Host genotypes can be combined with microbial DNA sequencing to predict performance traits or traits related to environmental impact, such as enteric methane emissions. Metagenome profiles were generated from 3139 rumen samples, collected from 1200 dual purpose ewes, using restriction enzyme-reduced representation sequencing (RE-RRS). Phenotypes were available for methane (CH4) and carbon dioxide (CO2) emissions, the ratio of CH4 to CH4 plus CO2 (CH4Ratio), feed efficiency (residual feed intake: RFI), liveweight at the time of methane collection (LW), liveweight at 8 months (LW8), fleece weight at 12 months (FW12) and parasite resistance measured by faecal egg count (FEC1). We estimated the proportion of phenotypic variance explained by host genetics and the rumen microbiome, as well as prediction accuracies for each of these traits.

**Results:**

Incorporating metagenome profiles increased the variance explained and prediction accuracy compared to fitting only genomics for all traits except for CO2 emissions when animals were on a grass diet. Combining the metagenome profile with host genotype from lambs explained more than 70% of the variation in methane emissions and residual feed intake. Predictions were generally more accurate when incorporating metagenome profiles compared to genetics alone, even when considering profiles collected at different ages (lamb vs adult), or on different feeds (grass vs lucerne pellet). A reference-free approach to metagenome profiling performed better than metagenome profiles that were restricted to capturing genera from a reference database. We hypothesise that our reference-free approach is likely to outperform other reference-based approaches such as 16S rRNA gene sequencing for use in prediction of individual animal performance.

**Conclusions:**

This paper shows the potential of using RE-RRS as a low-cost, high-throughput approach for generating metagenome profiles on thousands of animals for improved prediction of economically and environmentally important traits. A reference-free approach using a microbial relationship matrix from log_10_ proportions of each tag normalized within cohort (i.e., the group of animals sampled at the same time) is recommended for future predictions using RE-RRS metagenome profiles.

**Supplementary Information:**

The online version contains supplementary material available at 10.1186/s12711-023-00822-1.

## Background

Animal protein is an important element of human nutrition [1]. However, the sustainability of animal agriculture depends on our ability to produce sufficient levels of energy-rich animal protein to meet the demands of population growth, while mitigating its environmental impact (e.g., greenhouse gas emissions). The impact of livestock production on greenhouse gas emissions is particularly evident in countries, such as New Zealand that have a large pastoral sector, where 35% of national greenhouse gas emissions can be attributed to enteric fermentation in livestock [2]. To mitigate this impact, livestock production on the global stage is shifting from a production-oriented model to a sustainability model, which aims at considering not only the efficiency of the animal for production but also its impact on the environment.

Animal breeding programs have shown promise in our ability to select for animals that have reduced greenhouse gas emissions, and therefore a lower environmental impact [3, 4]. However, sustainable livestock production must also account for and minimise feed intake per unit of product (feed efficiency). Given that methane emissions are a result of digestion of feed, the two are inextricably linked. Therefore, the genetic relationship between feed efficiency and greenhouse gas emissions is an important consideration when developing tools to select for animals that have a lower impact on the environment and have fewer associated costs to the producer [5].

While genetic selection for reduced greenhouse gas emissions using direct measures is promising, there are other biological mechanisms that may more accurately identify individuals with a propensity to emit lower levels of greenhouse gases. One such tool is metagenomics. The rumen microbiome is composed of microbes that partially ferment the feed ingested by the animal. These microbes are crucial for making the nutrients of those feedstuffs available to the animal, but the methane formed as a by-product is emitted into the atmosphere as a greenhouse gas. It has been shown that specific microbes, as well as the entire microbial profile, can be associated with both greenhouse gas emissions and feed efficiency [6–10], with a significant proportion of phenotypic variation explained by relationships between individual rumen microbiomes, often referred to as microbiability [11]. Furthermore, these microbes and profiles have been shown to be heritable [12–15], suggesting that sustained progress can be achieved through selection practices. However, previous studies have typically been on a small scale and used technologies that either do not scale well when considering implementation in industry (e.g., whole-genome sequencing) or do not capture the breadth of diversity in the rumen (i.e., prokaryotic 16S rRNA gene sequencing) [16]. Recently, Hess et al. [17] developed a restriction enzyme-reduced representation sequencing (RE-RRS) approach that overcomes the shortcomings of other technologies to harness metagenomic information that can be used at the production level, due to its low cost and potential for high-throughput.

The value of metagenomics to the industry is not only in the ability to produce information-rich data at low cost, but also the ability for the tool to be predictive in different environments. Metagenome profiles are influenced by environment [14] and so an assessment needs to be made about whether decisions based on the metagenome profile of an animal in one environment will be stable across other environments. Furthermore, while greenhouse gas emissions and feed efficiency are obvious choices for the involvement of the metagenome, the value of harnessing metagenomic information can be extended if there is an association with other, perhaps less obvious, production traits.

The aim of this study was to investigate the impact of rumen metagenome profiles on greenhouse gas, feed efficiency, production, and health traits in sheep. This involved evaluating: (1) different methods for generating a microbial relationship matrix, (2) performance when using metagenome profiles collected at the same time as the phenotype was collected, and (3) performance when using metagenome profiles collected at a different time than the phenotype, representing differences in age and diet. Performance was evaluated by estimating the microbiability (the proportion of the phenotypic variance that was explained by the metagenome profiles), and prediction accuracy (the correlation between the phenotype adjusted for fixed effects and the prediction from genomics, metagenome profile or the combination of the two). This work is essential when considering the value of capturing metagenomic information in a practical agricultural setting.

## Methods

The animal experiments conducted adhered to the guidelines of the 1999 New Zealand Animal Welfare Act and AgResearch Code of Ethical Conduct. The trials of the current study were approved by the AgResearch Invermay (Mosgiel, NZ) Animal Ethics committee with the approval numbers: 13081, 13419, 13563, 13742, 13892, 14055, 14066, and 14221.

### Animals

The dual-purpose composite ewes used in this study were involved in a variety of feed intake [5] and methane [18] trials at the Invermay (Mosgiel) campus, and Woodlands farm, of AgResearch in New Zealand. Animals were born between 2014 and 2016 from three New Zealand flocks: one AgResearch flock (Flock 2638), one selection line flock containing sheep selected for high or low methane yield [19] (Flock 3633), and one Central Progeny Test flock (Flock 4640). Metagenome profiles were generated from rumen samples that were collected from the sheep at various time points throughout their life [14]. The metagenome profiles included in this study (Table 1) included samples taken when the sheep were fed two different diets: lucerne pellets (lucerne) and New Zealand ryegrass-based pasture (grass); and were classified as two age groups: Lambs (less than 15 months old) and adults (greater than 15 months old). The combination of feed and age resulted in three groups that were evaluated separately in this study: grass lamb, grass adult and lucerne lamb, corresponding to the GLS, GAS and LLS groups in Hess et al. [14]. All rumen samples were taken from animals when they were fed ad libitum and had been off feed for 2 to 4 h (i.e. “Short” time off feed in [14]). Across the 1200 sheep, 118 sires were represented, with each sire having 10 ± 5 offspring. In total, 939 dams were represented, with each dam having 1.3 ± 0.6 offspring.

**Table 1:** Number of animals with phenotypes recorded for each trait by rumen sample group

Trait		Grass lamb	Grass adult	Lucerne lamb
Methane-related	Methane (CH4, g/day)	1051	1010	958
	Methane ratio (CH4Ratio, mM/Mol)	1051	1010	958
	Carbon dioxide (CO2, g/day)	1051	1010	958
	Liveweight (LW, kg)^a^	1051	1010	958
Feed intake	Residual feed intake (RFI, MJ/day)	0	0	984
Production and health	8-month liveweight (LW8, kg)	1074	1080	985
	12-month fleece weight (FW12, kg)	1029	1076	980
	Faecal egg count (FEC1, epg)	967	961	866
Total rumen samples		1074	1080	985

^a^Liveweight taken at the same time as the portable accumulation chamber measurement and rumen sample collection

*epg* eggs per gram

### Genotypes

Animals were sequenced on a variety of Illumina beadchips, as shown in Table 2, and imputation was used to get high-density single nucleotide polymorphism (SNP) genotypes on all animals. We capitalized on the strong links between these flocks and the New Zealand sheep population [3, 20], and included genotypes on other New Zealand sheep to improve imputation accuracy. For each genotype panel, SNPs were filtered to retain markers where: both SNP probes uniquely mapped to the *Ovis aries* (OAR)v3.1 genome reference, one SNP probe mapped with zero mismatches, no indels were found, and both SNP probes were in the same orientation and position with exactly one mismatch (the target SNP). Subsequently, each genotype panel was imputed using the Beagle v5.1 software [21] from the SNPs that were overlapping with the high-density (HD) panel to retain 568,142 HD SNPs. Beagle’s default settings were used except for effective population size (ne) which was set to 500 to account for the diversity across sheep breeds. The phased and imputed sets were then subset to only include the individuals in this study, which were used to construct a genomic relationship matrix (GRM), using method 1 of VanRaden [22].

**Table 2:** Number of animals and SNPs genotyped on each SNP panel and used for imputation

Illumina SNP panel	Number of SNPs	Number of animals genotyped	Number of SNPs for imputation	Number of animals in this study	Imputation accuracy
AgR Custom 5k LD	5283	17,631	3885	123	0.86 ± 0.20
AgR Custom 6k LD	6015	8798	4206	9	0.85 ± 0.20
ISGC 15K	15,000	25,831	11,799	612	0.92 ± 0.20
AgR Custom 18k LD v2A1	16,399	24,912	12,266	24	0.92 ± 0.20
AgR Custom 18k LD v2C1	16,227	4695	12,478	3	0.88 ± 0.20
ISGC Ovine HD	606,006	22,802	568,142	429	NA

Imputation accuracy is reported as the average SNP-based R^2^ from BEAGLE for the full dataset

### Rumen sample collection and metagenome profiling

This study used 3139 (Table 1) of the 4479 rumen metagenome profiles that were generated by Hess et al. [14]. These samples are described in Additional file 1: Tables S1a, S1b and S1c, and were from the GLS (grass lamb: samples from lambs taken 2–4 h after removal from pasture), GAS (grass adult: samples from adults taken 2–4 h after removal from pasture) and LLS (lucerne lamb: samples from lambs taken 2–4 h after eating lucerne pellets ad libitum) groups reported by Hess et al. [14]. Briefly, 30 mL rumen contents were sampled via stomach intubation and stored at − 20 °C until freeze drying. The set of animals that were from the same flock and birth year and were measured over the same ~ 2-day period were considered to be the same cohort for rumen sampling. Samples were freeze-dried for one week in a CHRIST Gamma 1-16 LSCplus freeze dryer then ground to a powder using a Magic Bullet kitchen blender (NutriBullet New Zealand, Auckland, New Zealand) with a custom-made cup. Cups were cleaned in a laboratory dishwasher between samples to avoid cross-contamination. DNA was extracted using a combined beat-beating, phenol and column purification protocol using the QIAquick 96 PCR purification kit (Qiagen, Hilden, Germany), then sequenced using RE-RRS with the *Pst*I restriction enzyme, as described by Hess et al. [17]. Sequencing was performed with 384 samples per lane of sequencing on an Illumina HiSeq2500 machine, generating 101 bp single end reads using version 4 chemistry.

#### Sequence processing

Demultiplexing and trimming were carried out using the GBSX [23] and Cutadapt [24] packages. GBSX was run using default parameters except that no mismatches were allowed in the barcode or cut site. Cutadapt was run with a Phred quality score threshold of 20 and a minimum length of 40 bp. Samples with fewer than 100,000 reads after quality control were considered failed samples. Sequences were run through both the reference-based (RB) and the reference-free (RF) pipelines, as briefly described below, with further details reported by Hess et al. [17]. Both pipelines produce a table of counts with one row per sample and one column per microbial genus (RB) or tag (RF).

#### Reference-based (RB)

The RB pipeline used nucleotide BLAST (task = blastn, word size = 16, e-value = 0.01) in BLAST v2.2.28 + [25] to compare sequences against microbial genome assemblies from the Hungate 1000 Collection [26], with the addition of four *Quinella* genome assemblies [27]. Taxonomy of sequences was assigned using an R implementation of the algorithm from MEGAN [28] with default parameters. The microbiome profile for each sample was the number of sequences assigned at the genus level for each of the 60 genera represented in the Hungate1000 Collection, plus *Quinella*.

#### Reference free (RF)

The RF pipeline generates a set of “tags”: non-redundant 65-bp long sequences that start at the initial cut site and are observed in at least 25% of samples. The metagenome profile contains the number of times each tag is observed in each sample. The tags used in our study were generated separately within each group (grass lamb, grass adult and lucerne lamb).

#### Metagenome relationship matrices

Metagenome relationship matrices (MRM) were first developed by Ross et al. [29] and have become the standard method for integrating metagenome profiles into prediction equations in livestock [16]. In our study, MRM were generated separately for each group. Counts for each of the metagenome profiles were transformed to the log_10_ proportion of assigned reads by adding one to each count and dividing by the row sum (i.e., total number of reads accounted for in the profile for that sample plus the number of columns) then taking the log (base 10). These logged proportions were then normalized in two ways:

1. L10: each column was normalized, such that each column had a mean of 0 and a standard deviation of 1, i.e., in matrix notation from the proportion matrix $\mathbf{P}$, individual $i$ for genus/tag $j$:
  1 $${x}_{ij}=\frac{{log}_{10}\left({p}_{ij}\right)-{\mu }_{{log}_{10}\left({p}_{.j}\right)}}{{\sigma }_{{log}_{10}\left({p}_{.j}\right)}}.$$
2. CA: each column was normalized within rumen sample cohort, such that each cohort had a mean of 0 and standard deviation of 1 for each genus or tag, i.e., in matrix notation from the proportion matrix $\mathbf{P}$, individual $i$ in cohort $c$ for genus/tag $j$:
  2 $${x}_{{i}_{c}j}=\frac{{log}_{10}\left({p}_{{i}_{c}j}\right)-{\mu }_{{log}_{10}\left({p}_{{.}_{c}j}\right)}}{{\sigma }_{{log}_{10}\left({p}_{{.}_{c}j}\right)}}.$$

The subsequent matrices (with dimension number of samples by number of genera/tags) were used to generate a metagenome relationship matrix (MRM) by taking the correlation of the transpose of the matrix (${\mathbf{X}}^{\mathrm{T}}$) and using the cor function in R to generate an n × n matrix where n is the number of samples. This approach generated four MRM for each group: RBL10, RBCA, RFL10, and RFCA, representing the RB and RF profiles for L10 and CA normalization methods.

### Phenotypes

#### Methane-related traits

Measures of methane, carbon dioxide and oxygen were made using a system with 10 portable accumulation chambers (PAC) as described by Jonker et al. [18]. Phenotypes for the lamb groups were collected when individuals were 6 to 13 months old, and phenotypes for the adult groups were collected when individuals were 15–18 months old. In brief, animals were allocated to a time period and chamber using a randomized incomplete block design with allocation to a measurement “lot” of 10 or 12 animals randomly within sire. Allocation to a chamber was random within the lot. Four lots were removed from feed at a given time, with all animals off feed for a minimum of one hour before they were placed in the individual chambers. Liveweights and time off feed were recorded. Methane (CH_4_), carbon dioxide (CO_2_) and oxygen (O_2_) in grams per day were estimated from a one-hour measure in the chambers. Methane ratio was calculated as CH_4_/(CO_2_ + CH_4_), expressed as mM/Mol, and is commonly used as a substitute for methane yield when feed intake data is not available. Gas measures were scaled within lot to account for time off feed by dividing an individual’s measurement by the mean of the lot and multiplying it by the overall population mean [18]. The methane-related traits evaluated in our study were scaled CH_4_ (CH4), scaled CO_2_ (CO2), scaled methane ratio (CH4Ratio) and liveweight at the time of rumen sample collection (LW). Trait units are in Table 1. Although Jonker et al. [18] describe two gas measurement sessions ~ 14 days apart, only the measurement taken on the same day that the rumen sample was collected were considered for this study.

#### Residual feed intake

Residual feed intake was measured at AgResearch’s Invermay campus, as described by Johnson et al. [5]. In brief, over three years, five cohorts of approximately 200 lambs had individual feed intakes measured through a feed intake facility. The animals were sourced from Flocks 2638, 3633 and 4640 and were approximately nine to 12 months old at the time of measurement. The animals were transitioned to a lucerne pellet diet sourced from J.T. Johnson & Sons (Kapunda, South Australia, Australia, www.jtj.com) over a period of two weeks. Following the introduction period, daily intake data was collected for approximately 42 consecutive days. The weight of the animals was measured twice weekly and was used to estimate the growth rate of the animals over the 42 days. The resulting feed intake, weight and growth rate data were used to estimate the residual feed intake (RFI) trait, which is the residual of a model where intake is fitted as the dependent variable with metabolic weight, growth rate, cohort, flock and pen fitted as explanatory variables as described in Johnson et al. [5] based on Koch et al. [30]. Methane traits (CH4, CO2, CH4Ratio and LW) were collected on these individuals during these trials, using the approach described in the “Methods” section entitled ‘Methane-related traits’.

#### Health and production traits

Three industry-recorded traits (Sheep Improvement Ltd, SIL; www.sil.co.nz) were selected to evaluate the impact of including rumen metagenome profiles for prediction of economically important health and production traits. These juvenile traits were liveweight at 8 months (LW8, kg), summer strongyle faecal egg count (FEC1, epg) and fleece weight at 12 months of age (FW12, kg). Data were transformed or scaled according to standard SIL protocols on the full dataset [31], which was then reduced to the set of 1200 animals that had rumen metagenome profiles (Table 1). Samples for FEC1 were collected according to the WormFEC protocol [32] developed by AgResearch Ltd., whereby lambs were treated with an anthelmintic at weaning, and then individually sampled once the mob (set of sheep grazed together in the period prior to data collection) faecal egg count reaches 800 eggs per gram (epg). Faecal egg counts had 50 added to them then the log (base e) was taken to get FEC1.

### Parameter estimation and prediction accuracy

#### Parameter estimation

Models used in our study were run in ASReml 4.1 [33] and reflected those used in previous analyses of these traits in expanded datasets, with any variables that did not have variation within this dataset removed (e.g. sex because all individuals in this study were female):

3 $$CH4 = \mu +brr+aod+bdev+cg4+animal+metagenome+e,$$

4 $$CH4Ratio = \mu +brr+aod+bdev+cg4+animal+metagenome+e,$$

5 $$CO2= \mu +brr+aod+bdev+cg4+animal+metagenome+e,$$

6 $$LW= \mu +brr+aod+bdev+cg4+animal+metagenome+e,$$

7 $$RFI= \mu +brr+aod+bdev+cg4+animal+metagenome+e,$$

8 $$LW8 = \mu +brr+aod+bdev+flock.cgLW8+animal+metagenome+e,$$

9 $$FEC1= \mu +bdev+flock.cgFEC+animal+metagenome+e,$$

10 $$FW12= \mu +brr+aod+bdev+flock.cgFW12+animal+metagenome+e,$$

where $\mu$ is the overall mean, $brr$ is the fixed class of birth and rearing rank (combination of birth rank i.e. born as a single, twin or triplet; and rearing rank i.e. reared as a single, twin or triplet), $aod$ is the fixed class of age of dam when the individual was born (3 levels), $bdev$ is the fixed covariate of the birth date deviation from the mean for that flock within that year. Contemporary groups ($cg$) were fitted as fixed class effects: $cg4$ is the combination of flock and birth year, $cgLW8$ is the combination of birth year, LW8 mob and weaning weight mob; $cgFEC$ is the combination of birth year and FEC1 grazing mob; and $cgFW12$ is the combination of birth year, FW12 mob, LW8 mob and WWT mob. The random animal genetic effect was assumed to have a mean of 0 and a variance of $\mathbf{G}{\sigma }_{g}^{2}$ where $\mathbf{G}$ is the GRM and ${\sigma }_{g}^{2}$ is the genetic variance. The random metagenome effect was assumed to have a mean of zero and a variance of $\mathbf{M}{\sigma }_{m}^{2}$ where $\mathbf{M}$ is the MRM and ${\sigma }_{m}^{2}$ is the metagenomic variance. The animal genetic and metagenome effects were assumed to be independent. The residual ($e$) was assumed to have a mean of 0 and variance of $\mathbf{I}{\sigma }_{e}^{2}$ where $\mathbf{I}$ is an n × n identity matrix and ${\sigma }_{e}^{2}$ is the residual variance. The phenotypic variance was calculated as the sum of the animal genetic, metagenomic and residual variances. Heritability was estimated as the proportion of the phenotypic variance that was attributed to the random animal genetic effect. Likewise, microbiability was estimated as the proportion of phenotypic variance that was attributed to the metagenome effect. The estimated animal genetic effect for an individual ($\widehat{\mathrm{G}}$) is the breeding value (BV), and the estimated metagenome effect for a sample ($\widehat{\mathrm{M}}$) is the metagenome value (MV). The proportion of the phenotypic variance explained by both G and M was also estimated by dividing the sum of ${\sigma }_{g}^{2}$ and ${\sigma }_{m}^{2}$ by the phenotypic variance. The models described are referred to as the G + M model; they were additionally run fitting only the animal genetic effect (G; i.e., removing the metagenome effect) and only the metagenome effect (M; i.e., removing the animal genetic effect).

Methane-related traits were initially analysed using the metagenome profile taken at the same time they were recorded (Table 3). In addition, the grass lamb methane-related traits were also analysed using the grass adult metagenome profiles, and vice versa, to evaluate the ability of a metagenome profile taken at a different age to explain methane-related traits. RFI was collected only in the lucerne lamb group and was analysed using the lucerne lamb metagenome profiles as well as the grass adult and grass lamb metagenome profiles. Health and production traits were not collected at the same time as any of the rumen samples and were analysed using each of the metagenome profiles separately.

**Table 3:** Phenotypes and metagenome profiles analysed in this study

Traits analysed	Trait group	Metagenome profile	Tables with corresponding results
Methane-related	Grass lamb	Grass lamb	Table 4 and Additional file 2: Table S2
	Grass adult	Grass adult	Table 4 and Additional file 2: Table S2
	Lucerne lamb	Lucerne lamb	Table 4 and Additional file 2: Table S2
Methane-related	Grass lamb	Grass adult	Table 5
	Grass adult	Grass lamb	Table 5
Residual feed intake	Lucerne lamb	Lucerne lamb	Table 6
	Lucerne lamb	Grass lamb	Table 6
	Lucerne lamb	Grass adult	Table 6
Health and production	Other	Grass lamb	Table 7 and Additional file 3: Table S3
	Other	Grass adult	Table 7 and Additional file 3: Table S3
	Other	Lucerne lamb	Table 7 and Additional file 3: Table S3

#### Prediction accuracy

The estimation of prediction accuracy and bias used the same models as described in the “Methods” subsection entitled ‘Parameter estimation’, and used cross-fold validation, whereby each rumen sample cohort was a different fold. Prediction accuracy was estimated as the correlation between the MV or BV from the fold where the corresponding phenotype was omitted, and the phenotype adjusted for fixed effects. Prediction accuracy for the BV was not divided by the square root of heritability because we wanted to evaluate the accuracy of predicting the phenotype rather than the BV, so we could compare the prediction accuracy of the BV and MV given that MV may capture more than just the genetics of the individual. The regression coefficient of the adjusted phenotype on the MV or BV was an indicator of bias, with unbiased prediction being equal to 1. Given that each cohort had a slightly different size, the mean and standard error of accuracy and bias were represented by the weighted mean and weighted standard deviation across the cohorts, with weights equal to the size of the cohort. Weighted paired t-tests were performed with α = 0.05 to test the hypotheses that: (1) prediction accuracy of MV was greater than that of BV; (2) the regression coefficient of the adjusted phenotype on the MV was not equal to the regression coefficient of the adjusted phenotype on the BV; and (3) the absolute bias of the MV was smaller than that of the BV.

## Results

### Methane-related traits and metagenome profiles

#### Methods for generating MRM

The method used to generate an MRM could have a large impact on the microbiability and the ability of the MRM to accurately predict individual performance for a trait. We explored two methods of metagenome profiling: RB and RF; and two methods of normalization: with and without a cohort-specific adjustment. We evaluated the performance of each of these methods using a model that captures the metagenomic relationship between samples, ignoring the genomic relationship between samples.

**Fig. 1:**
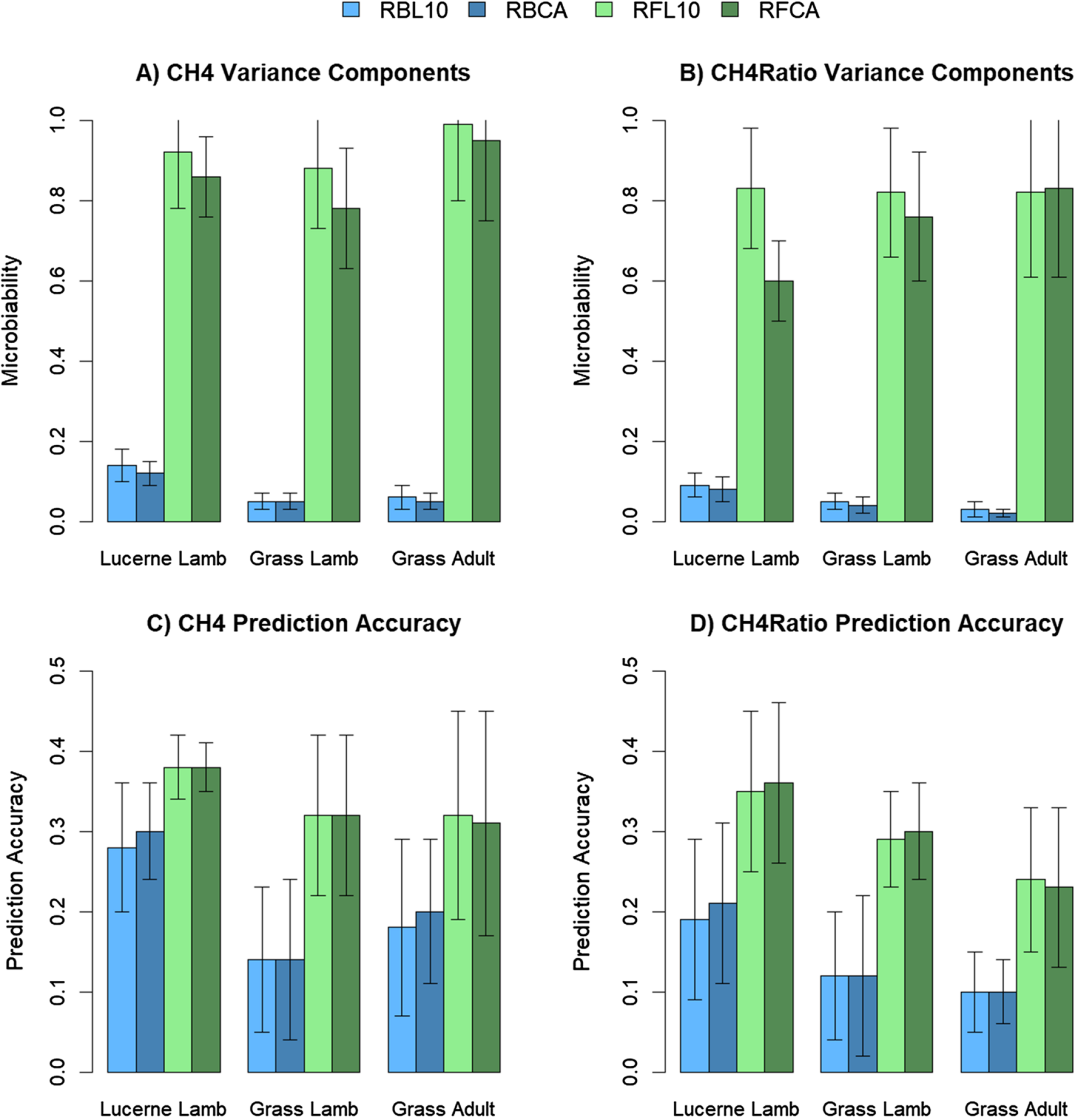
Variance components (**A** and **B**) and prediction accuracies (**C** and **D**) for methane emissions (**A** and **C**) and methane ratio (**B** and **D**) using different MRM. Variance components and prediction accuracies were estimated using models capturing the metagenomic relationship between samples calculated in four different ways: using Reference Based (RB) or Reference Free (RF) profiles that were each adjusted by taking the log (base 10) of proportions followed by normalization (L10) or by taking the log (base 10) of proportions followed by normalization within cohort (CA). The traits methane emissions (CH4) or methane ratio (CH4Ratio) were analyzed in three datasets: lambs fed lucerne pellets (lucerne lamb), lambs grazing pasture (grass lamb) and adults grazing pasture (grass adult). Prediction accuracy was estimated using cross-fold validation with each cohort a different fold

Microbiability estimates ranged from 0.00 (CO2: grass adult and grass lamb) to 0.14 (CH4: lucerne lamb) when using the RB MRM and from 0.00 (CO2: grass lamb) to 0.99 (LW8: lucerne lamb) when using the RF MRM (Fig. 1) and (see Additional file 2: Table S2). All traits had microbiability estimates significantly different from 0 (p < 0.05) for at least one of the MRM, except for CO2 in the two groups on grass, and LW in the grass adult (see Additional file 2: Table S2). Microbiability estimates using the RF MRM were much larger than those using the RB MRM (Fig. 1) and (see Additional file 2: Table S2), while the difference in microbiability estimates when using the L10 and CA MRM was very small. Some models using the RFL10 MRM had convergence or singularity issues, indicating that using the RFCA MRM may result in more robust models (see Additional file 2: Table S2). Prediction accuracy (the correlation between the MV and adjusted phenotype when using cross-fold validation) was also higher when using the RF MRM compared to the RB MRM, and there was little difference in prediction accuracy between the L10 and CA MRM, consistent with findings from evaluating the microbiability (Fig. 1) and (see Additional file 2: Table S2).

These results highlight the importance of the additional information captured by the RF approach compared to the RB approach. The RFCA approach is the most promising and reliable approach for trait predictions using MRM among those we evaluated (Fig. 1) and (see Additional file 2: Table S2), and the remainder of the paper will focus on this method for generating the MRM.

#### Genomes vs. rumen metagenomes

Table 4 gives the heritability, microbiability and prediction accuracy estimates for the methane-related traits when using the RFCA MRM when fitting only G, only M, or G + M models. The results for methane emissions (CH4) and methane ratio (CH4Ratio) showed a similar pattern, whereby the microbiability estimates were much higher than the heritability estimates within each group; and this was true whether fitting models with only G or only M, as well as the G + M models. Consistent with these results, prediction accuracy was higher when fitting a model that considered metagenomic (M) rather than genomic (G) relationships. Both heritability and microbiability estimates were reduced in the G + M models (G_G+M_ and M_G+M_) compared to the models fitting only G (G_G_) or only M (M_M_), indicating that some of the variance can be captured by both G and M and is being partitioned to one or the other in the G + M model. The best prediction accuracy estimates were obtained from the G + M model. These prediction accuracies were not always higher than the models fitting only M (M_M_) but were consistently higher than those fitting only G (G_G_). The BV accuracy did not increase in the G + M model (G_G+M_) compared to the G model (G_G_), which indicates that incorporating metagenome profiles does not improve our ability to predict BV.

**Table 4:** Variance components and prediction accuracies for methane-related traits

Phenotype	Model^a^	Component^b^	Lucerne lamb		Grass lamb		Grass adult	
			PropVar^c^	Accuracy^d^	PropVar^c^	Accuracy^d^	PropVar^c^	Accuracy^d^
CH4	G	G_G_	0.35 ± 0.08	0.11 ± 0.05	0.25 ± 0.07	0.13 ± 0.10	0.33 ± 0.07	0.18 ± 0.08
	M	M_M_	0.86 ± 0.10	0.38 ± 0.03	0.78 ± 0.15	0.32 ± 0.10	0.95 ± 0.20	0.31 ± 0.14
	G + M	G_G+M_	0.22 ± 0.06	0.08 ± 0.06	0.18 ± 0.06	0.13 ± 0.14^f^	0.25 ± 0.11^f^	0.17 ± 0.08^f^
		M_G+M_	0.78 ± 0.06	0.38 ± 0.03	0.71 ± 0.15	0.32 ± 0.10^f^	0.75 ± 0.11^f^	0.31 ± 0.14^f^
		G + M_G+M_	1.00 ± 0.00	0.38 ± 0.03	0.89 ± 0.15	0.34 ± 0.09^f^	1.00 ± 0.00^f^	0.35 ± 0.12^f^
CH4Ratio	G	G_G_	0.35 ± 0.07	0.18 ± 0.04	0.24 ± 0.07	0.10 ± 0.06	0.33 ± 0.07	0.17 ± 0.18
	M	M_M_	0.60 ± 0.10	0.36 ± 0.10	0.76 ± 0.16	0.30 ± 0.06	0.83 ± 0.22	0.23 ± 0.10
	G + M	G_G+M_	0.25 ± 0.07	0.15 ± 0.03	0.21 ± 0.06	0.09 ± 0.05^f^	0.28 ± 0.07	0.17 ± 0.17
		M_G+M_	0.53 ± 0.10	0.36 ± 0.10	0.67 ± 0.15	0.30 ± 0.07^f^	0.59 ± 0.21	0.22 ± 0.10
		G + M_G+M_	0.78 ± 0.11	0.37 ± 0.07	0.88 ± 0.16	0.30 ± 0.05^f^	0.86 ± 0.20	0.24 ± 0.14
CO2	G	G_G_	0.27 ± 0.07	0.14 ± 0.08	0.19 ± 0.06	0.18 ± 0.13	0.38 ± 0.07	0.24 ± 0.12
	M	M_M_	0.46 ± 0.12	0.24 ± 0.11	0.00 ± 0.00	− 0.05 ± 0.09	0.14 ± 0.13	0.05 ± 0.09
	G + M	G_G+M_	0.27 ± 0.08	0.13 ± 0.08	0.19 ± 0.06	0.18 ± 0.13	0.37 ± 0.07	0.24 ± 0.12
		M_G+M_	0.42 ± 0.11	0.24 ± 0.11	0.00 ± 0.00	− 0.05 ± 0.10	0.05 ± 0.08	0.04 ± 0.10
		G + M_G+M_	0.69 ± 0.13	0.27 ± 0.09	0.19 ± 0.06	0.15 ± 0.10	0.42 ± 0.11	0.24 ± 0.13
LW	G	G_G_	0.45 ± 0.08	0.19 ± 0.06	0.33 ± 0.07	0.14 ± 0.06	0.44 ± 0.07	0.22 ± 0.09
	M	M_M_	0.33 ± 0.09	0.26 ± 0.04	0.45 ± 0.14	0.22 ± 0.10	0.88 ± 0.21	0.26 ± 0.11
	G + M	G_G+M_	0.42 ± 0.08	0.19 ± 0.06	0.33 ± 0.07	0.16 ± 0.06	0.41 ± 0.11^e,f^	0.23 ± 0.09^f^
		M_G+M_	0.26 ± 0.08	0.25 ± 0.05	0.42 ± 0.13	0.22 ± 0.10	0.59 ± 0.11^e,f^	0.25 ± 0.11^f^
		G + M_G+M_	0.68 ± 0.10	0.28 ± 0.06	0.76 ± 0.13	0.28 ± 0.07	1.00 ± 0.00^e,f^	0.32 ± 0.06^f^

CH4 = scaled methane emissions; CH4Ratio = scaled methane ratio (CH_4_/(CH_4_ + CO_2_)); CO2 = scaled carbon dioxide emissions; LW = liveweight at time of rumen sampling

^a^Model fitting the random effects of animal genetics (G), metagenome profile (M) or both (G + M)

^b^Large letter(s) represent the component (genomics = G; metagenome = M; or sum of the two = G + M), and the subscript represents the model used

^c^PropVar = Proportion of the phenotypic variance (adjusted for fixed effects) explained by the component

^d^Accuracy = Correlation between phenotype adjusted for fixed effects and the component when using cross-fold validation with each Cohort a different fold

^e^Run in ASReml 4.2 because ASReml 4.1 had major convergence issues

^f^At least one of the models contributing to this value had convergence or singularity issues

CO2 showed a different pattern to that of CH4 and CH4Ratio; whereby microbiability estimates were larger than heritability estimates for the lucerne lamb group but smaller for the two grass groups, with a microbiability estimate of zero for the grass lamb group (Table 4). There was little change in estimates of heritability and microbiability when fitting the G + M model compared to only G or only M, indicating that the genomic and metagenomic drivers of CO2 are largely independent. However, the microbiability estimate for the grass adult group was reduced when fitting the G + M model. Prediction accuracy was highest for the G + M component of the G + M model (G + M_G+M_) for the lucerne lamb group, but the G + M model did not result in prediction accuracy estimates higher than the model fitting only G (G_G_) for either of the grass groups. The prediction accuracy of the metagenome was not significantly different from 0 in any model for either of the grass groups. Given that the metagenome profiles did not explain a significant amount of the variation in CO2 in sheep on pasture, CO2 was not further evaluated for its ability to be predicted by metagenome profiles from a different group.

Microbiability estimates for liveweight at the time of rumen sampling (LW) were higher than the heritability estimates for the two grass groups, but smaller for the lucerne lamb group (Table 4). Prediction accuracy was at least as good for MV (M_M_ and M_G+M_) as BV (G_G_ and G_G+M_) and the highest accuracy estimate was the G + M component of the G + M model (G + M_G+M_). This estimate (G + M_G+M_) was double the accuracy of the BV estimate from the model fitting only G (G_G_) for the grass lamb group.

#### Using profiles collected at a different age

When using the metagenome profile from a different age than that from when the phenotype was collected (e.g., phenotype as a lamb and metagenome profile as an adult), microbiability estimates (Table 5) were smaller than when the metagenome profile was collected at the same time as the phenotype (Table 4) for all traits except LW in lambs (predicted from adult metagenome profiles). However, the microbiability estimates were typically at least as large as the corresponding heritability estimate (Table 5). In spite of some of the microbiability estimates being greater than the corresponding heritability estimate, the prediction accuracies tended to be very similar for predictions with G or M both when fitting G and M independently (i.e., G_G_ and M_M_) and when fitting them simultaneously (i.e., G_G+M_ and M_G+M_).

**Table 5:** Variance components and prediction accuracies for methane-related traits from metagenome profiles at different ages

Phenotype	Metagenome profile	Model^a^	Component^b^	Proportion of variance^c^	Accuracy^d^	Regression coefficient^e^
Grass adult CH4	Grass lamb	G	G_G_	0.32 ± 0.07	0.16 ± 0.12	0.76 ± 0.57
		M	M_M_	0.52 ± 0.17	0.22 ± 0.09	1.00 ± 0.37
		G + M	G_G+M_	0.31 ± 0.07	0.16 ± 0.11	0.75 ± 0.56
			M_G+M_	0.47 ± 0.16	0.22 ± 0.08	1.09 ± 0.37
			G + M_G+M_	0.78 ± 0.17	0.28 ± 0.12	1.01 ± 0.41
Grass lamb CH4	Grass adult	G	G_G_	0.30 ± 0.08	0.13 ± 0.10	0.78 ± 0.61
		M	M_M_	0.52 ± 0.21	0.16 ± 0.10	1.03 ± 0.69
		G + M	G_G+M_	0.27 ± 0.08	0.12 ± 0.12	0.78 ± 0.80
			M_G+M_	0.45 ± 0.20	0.15 ± 0.09	1.14 ± 0.72
			G + M_G+M_	0.71 ± 0.21	0.18 ± 0.09	0.86 ± 0.28
Grass adult CH4Ratio	Grass lamb	G	G_G_	0.35 ± 0.08	0.16 ± 0.17	0.82 ± 0.82
		M	M_M_	0.31 ± 0.15	0.14 ± 0.10	0.91 ± 0.62
		G + M	G_G+M_	0.34 ± 0.08	0.17 ± 0.16	0.90 ± 0.84
			M_G+M_	0.21 ± 0.12	0.14 ± 0.09	1.21 ± 0.78
			G + M_G+M_	0.55 ± 0.14	0.20 ± 0.13	0.86 ± 0.50
Grass lamb CH4Ratio	Grass adult	G	G_G_	0.28 ± 0.07	0.14 ± 0.07	0.92 ± 0.58
		M	M_M_	0.37 ± 0.18	0.13 ± 0.11	1.30 ± 1.48
		G + M	G_G+M_	0.26 ± 0.07	0.13 ± 0.07	0.92 ± 0.59
			M_G+M_	0.18 ± 0.15	0.11 ± 0.11	2.89 ± 3.98
			G + M_G+M_	0.43 ± 0.16	0.15 ± 0.09	0.97 ± 0.72
Grass adult LW	Grass lamb	G	G_G_	0.41 ± 0.08	0.20 ± 0.08	0.84 ± 0.42
		M	M_M_	0.34 ± 0.15	0.17 ± 0.11	1.32 ± 1.00
		G + M	G_G+M_	0.39 ± 0.08	0.20 ± 0.09	0.84 ± 0.45
			M_G+M_	0.28 ± 0.14	0.16 ± 0.09	1.35 ± 0.95
			G + M_G+M_	0.67 ± 0.15	0.25 ± 0.07	0.91 ± 0.36
Grass lamb LW	Grass adult	G	G_G_	0.37 ± 0.08	0.17 ± 0.07	0.71 ± 0.36
		M	M_M_	0.79 ± 0.24	0.20 ± 0.06	1.21 ± 0.67
		G + M	G_G+M_	0.34 ± 0.07	0.16 ± 0.08	0.71 ± 0.38
			M_G+M_	0.56 ± 0.22	0.19 ± 0.06	1.49 ± 0.79
			G + M_G+M_	0.90 ± 0.21	0.23 ± 0.07	0.85 ± 0.36

CH4 = scaled methane emissions; CH4Ratio = scaled methane ratio (CH_4_/(CH_4_ + CO_2_)); CO2 = scaled carbon dioxide emissions; LW = liveweight at time of rumen sampling

^a^Model fitting the random effects of animal genetics (G), metagenome profile (M) or both (G + M)

^b^Large letter(s) represent the component (genomics = G; metagenome = M; or sum of the two = G + M), and the subscript represents the model used

^c^Proportion of the phenotypic variance (adjusted for fixed effects) explained by the component

^d^Correlation between phenotype adjusted for fixed effects and the component when using cross-fold validation

^e^Regression coefficient of phenotype adjusted for fixed effects on either breeding value (G), metagenome value (M) or the sum of both (G + M) when using cross-fold validation with each Cohort a different fold

The combined proportion of the total variance explained by BV_G+M_ + MV_G+M_ from the G + M models was always lower than for BV_G_ + MV_M_, indicating that there was some variation in each trait that was captured by both animal genetics and metagenome profile (Table 5). This leads to the heritability and microbiability estimates for G + M being slightly lower than from the models fitting only G or only M. However, despite explaining slightly less of the phenotypic variation, the prediction accuracies of BV_G+M_ and MV_G+M_ were very similar to those of BV_G_ and MV_M_, respectively.

Incorporation of metagenome profiles from samples collected at a different age into the normal prediction equations for these phenotypes (i.e., G + M model) increased prediction accuracy between 9% (using adult metagenome profiles to predict lamb CH4Ratio) and 38% (using adult metagenome profiles to predict lamb CH4) compared to just fitting the animal genetic random effect (Table 5). This improvement in prediction accuracy was significant (p = 0.03, paired t-test) when using adult metagenome profiles to predict lamb LW. These results indicate that metagenome profiles were predictive across time, can add more information than the animal genetic effect alone, and the improvement in prediction accuracy was not only observed when metagenome profiles were collected at the time of phenotype collection.

### Residual feed intake and metagenome profiles

#### Profiles from the same time point

The rumen samples for the lucerne lamb metagenome profiles were collected at the same time as feed intake was measured (Table 6). Microbiability estimates when using the lucerne lamb MRM were significantly greater than heritability estimates, and the combined proportion of phenotypic variance explained by G_G+M_ + M_G+M_ was almost 1. Prediction accuracy was also significantly greater when fitting metagenome profiles (M_M_ or M_G+M_) compared to fitting animal genetics (G_G_; p < 0.04, paired t-test). Prediction accuracy was highest when considering both the animal genetic and metagenome profile effects, but this prediction accuracy was only slightly higher than the MV alone (MV_M_ or MV_G+M_). Prediction bias was not significantly different between the models, and the regression coefficient was not significantly different to 1 (p > 0.05).

**Table 6:** Variance components and prediction accuracies for residual feed intake on lucerne pellets using genotypes and metagenomes

Metagenome profile	Model^a^	Component^b^	Proportion of variance^c^	Accuracy^d^	Regression coefficient^e^
Lucerne lamb	G	G_G_	0.39 ± 0.07	0.29 ± 0.08	1.06 ± 0.22
	M	M_M_	0.77 ± 0.07	0.47 ± 0.06	1.15 ± 0.52
	G + M	G_G+M_	0.30 ± 0.07	0.19 ± 0.13^f^	1.15 ± 0.63^f^
		M_G+M_	0.67 ± 0.07	0.46 ± 0.07^f^	1.20 ± 0.61^f^
		G + M_G+M_	0.98 ± 0.08	0.49 ± 0.06^f^	1.14 ± 0.52^f^
Grass lamb	G	G_G_	0.43 ± 0.08	0.29 ± 0.09	1.25 ± 0.48
	M	M_M_	0.74 ± 0.21	0.19 ± 0.05^f^	0.90 ± 0.44^f^
	G + M	G_G+M_	0.40 ± 0.08	0.28 ± 0.08^f^	1.28 ± 0.45^f^
		M_G+M_	0.57 ± 0.19	0.18 ± 0.06^f^	1.04 ± 0.56^f^
		G + M_G+M_	0.97 ± 0.19	0.31 ± 0.07^f^	1.05 ± 0.35^f^
Grass adult	G	G_G_	0.39 ± 0.07	0.28 ± 0.10	1.20 ± 0.50
	M	M_M_	0.69 ± 0.20	0.21 ± 0.16	1.08 ± 0.93
	G + M	G_G+M_	0.37 ± 0.07	0.27 ± 0.09^f^	1.27 ± 0.54^f^
		M_G+M_	0.48 ± 0.18	0.19 ± 0.16^f^	1.32 ± 1.14^f^
		G + M_G+M_	0.85 ± 0.19	0.31 ± 0.12^f^	1.16 ± 0.62^f^

^a^Model fitting the random effects of animal genetics (G), metagenome profile (M) or both (G + M)

^b^Large letter(s) represent the component (genomics = G; metagenome = M; or sum of the two = G + M), and the subscript represents the model used

^c^Proportion of the phenotypic variance (adjusted for fixed effects) explained by animal genetics (G, heritability), the metagenome profile (M, microbiability) or both (G + M)

^d^Correlation between phenotype adjusted for fixed effects and either breeding value (G), metagenome value (M) or the sum of both (G + M) when using cross-fold validation

^e^Regression coefficient of phenotype adjusted for fixed effects on either breeding value (G), metagenome value (M) or the sum of both (G + M) when using cross-fold validation with each Cohort a different fold

^f^At least one of the models contributing to this value had convergence or singularity issues

#### Profiles from different time points

Metagenome profiles from when the animals were on pasture (grass) as lambs and adults were also used to evaluate microbiability and prediction accuracy for RFI (Table 6). Microbiability estimates tended to be higher than the corresponding heritability estimates for grass lamb and grass adult metagenome profiles, with estimates for lambs being slightly larger than for adults. Microbiability estimates and MV accuracies using grass lamb or grass adult metagenome profiles were significantly different from zero (p < 0.05), and prediction accuracies were largest when using both animal genetics and metagenome profiles (G + M) to predict RFI, indicating that metagenome profiles from rumen samples taken when an animal is fed on pasture are predictive of RFI on a lucerne pellet diet.

### Health and production traits and metagenome profiles

Three health and production traits were evaluated for their microbiability and prediction accuracy when using metagenome profiles collected from the grass lamb, grass adult and lucerne lamb groups. These traits were liveweight at 8 months (LW8), summer strongyle faecal egg count (FEC1), and fleece weight at 12 months of age (FW12). Rumen samples were not collected at the same time as phenotype collection.

LW8 had similar heritability and microbiability estimates for G and M for both grass groups, as well as the same prediction accuracy estimates (Table 7). Fitting metagenome profiles for grass lambs or grass adults in addition to the animal genetic effect improved prediction accuracy, however, this improvement was not significant (p = 0.10, paired t-test). The proportion of phenotypic variation explained by G or M in the lucerne lamb group were higher than for the grass groups. The prediction accuracies for the G + M model (G + M_G+M_) using the lucerne lamb group were higher than for the grass groups, however, they also had larger standard errors and were therefore less reliable.

**Table 7:** Variance components and prediction accuracies for liveweight at eight months using genotypes and metagenomes

Metagenome profile	Model^a^	Component^b^	Proportion of variance^c^	Accuracy^d^	Regression coefficient^e^
Grass lamb	G	G_G_	0.35 ± 0.07	0.16 ± 0.11	0.67 ± 0.48
	M	M_M_	0.31 ± 0.13	0.16 ± 0.11	1.09 ± 0.76
	G + M	G_G+M_	0.35 ± 0.07	0.16 ± 0.11	0.68 ± 0.47
		M_G+M_	0.28 ± 0.12	0.16 ± 0.11	1.12 ± 0.80
		G + M_G+M_	0.63 ± 0.13	0.22 ± 0.11	0.81 ± 0.45
Grass adult	G	G_G_	0.36 ± 0.07	0.16 ± 0.13	0.66 ± 0.49
	M	M_M_	0.39 ± 0.18	0.16 ± 0.07	1.32 ± 0.76
	G + M	G_G+M_	0.34 ± 0.07	0.16 ± 0.12	0.67 ± 0.49
		M_G+M_	0.24 ± 0.15	0.15 ± 0.07	1.83 ± 1.10
		G + M_G+M_	0.58 ± 0.15	0.20 ± 0.11	0.78 ± 0.45
Lucerne lamb	G	G_G_	0.42 ± 0.08	0.18 ± 0.10	0.66 ± 0.36
	M	M_M_	0.43 ± 0.16	0.12 ± 0.02	0.78 ± 0.18
	G + M	G_G+M_	0.42 ± 0.08	0.36 ± 0.31	1.08 ± 0.57
		M_G+M_	0.45 ± 0.16	0.29 ± 0.37	1.17 ± 0.69
		G + M_G+M_	0.86 ± 0.17	0.41 ± 0.33	0.95 ± 0.23

^a^Model fitting the random effects of animal genetics (G), metagenome profile (M) or both (G + M)

^b^Large letter(s) represent the component (genomics = G; metagenome = M; or sum of the two = G + M), and the subscript represents the model used

^c^Proportion of the phenotypic variance (adjusted for fixed effects) explained by animal genetics (G, heritability), the metagenome profile (M, microbiability) or both (G + M)

^d^Correlation between phenotype adjusted for fixed effects and either breeding value (G), metagenome value (M) or the sum of both (G + M) when using cross-fold validation

^e^Regression coefficient of phenotype adjusted for fixed effects on either breeding value (G), metagenome value (M) or the sum of both (G + M) when using cross-fold validation with each Cohort a different fold

FEC1 had microbiability estimates of 0 and poor prediction accuracies with very high regression coefficients for metagenome profiles from both grass lamb and lucerne lamb groups (see Additional file 3: Table S3). The microbiability estimates for FEC1 when using metagenome profiles from the grass adult group were higher than the heritability estimates, and prediction accuracy was higher for MV than for BV, although not significantly. The highest prediction accuracy was obtained from the G + M model (G + M_G+M_), however, this prediction accuracy was very similar to the prediction accuracy when just M (M_M_) was fitted.

FW12 had similar results using each of the metagenome profiles (see Additional file 3: Table S3). The microbiability and prediction accuracy of M (M_M_ and M_G+M_) were slightly lower than for G. Consistent with findings for the other traits that we investigated, the prediction accuracy of G + M_G+M_ provided the highest prediction accuracy; however, the accuracy was only slightly higher than that from the model fitting only G. The highest prediction accuracy was from metagenome profiles from grass adults, followed by grass gambs and finally lucerne lambs.

These three traits show different examples of the potential for incorporating metagenome profiles to improve prediction of traits that are of importance to the New Zealand sheep industry.

## Discussion

### Animal genetic models

Genomic heritability estimates were significantly different from 0 for all traits investigated in this study and ranged from 0.19 (CO2 in lambs grazing on pasture) to 0.58 (FW12 in lambs and adults grazing on pasture; see Fig. 1, Tables 4, 5, 6 and 7 and see Additional file 2: Table S2 and Additional file 3: Table S3). Small fluctuations in heritability estimates for the same trait, e.g., FEC1 (see Additional file 3: Table S3), were due to slightly different individuals being included in each analysis, based on whether that individual had a metagenome profile for the group that was being evaluated. Heritability estimates were consistent with previously published estimates in the New Zealand sheep population for all traits. Jonker et al. [18] presented heritability estimates for methane emissions in New Zealand sheep from a larger dataset, of which the phenotypes from the grass diet in our study are a subset. Our heritability estimates are generally slightly higher than in Jonker et al. [18] (Table 4), but not significantly different. Apart from the difference in size in the datasets, other explanations for the slightly higher heritability estimates in our study are: (1) the heritability estimates reported by Jonker et al. [18] were obtained using pedigree-based relationships, rather than genomic relationships from high-density SNPs; and (2) Jonker et al. [18] had methane-related traits measured at multiple time points so they were able to fit a permanent environmental effect in their model, while in our study some of the permanent environmental effect of methane-related traits may have been captured by the animal genetic effect, inflating the heritability estimate. Johnson et al. [5] recently published the pedigree-based heritability estimate on the full set of sheep that have gone through the Feed Intake facility at AgResearch’s Invermay campus, and their estimate of 0.42 ± 0.09 is consistent with our genomic-based estimates of 0.39 to 0.43 (Table 6). Pickering et al. [31] estimated heritabilities for a number of traits of interest to New Zealand sheep breeders, including LW8 (> 1 M records), FEC1 (> 130k records) and FW12 (> 750k records). Our heritability estimates were reasonably consistent, although our estimate of heritability for FW12 was 0.58 (see Additional file 3: Table S3), compared to their estimate of 0.37. Our heritability estimates for all production and health traits were larger than those published in the studies mentioned, likely because our dataset was smaller, with ~ 1000 sheep and these sheep graze on research farms so management and level and species of parasite challenge is more consistent.

### Incorporating metagenome profiles: methane-related traits

We investigated four traits related to measuring the methane emission phenotype in this study: CH4, CH4Ratio, CO2 and LW. Three of these traits, CH4, CH4Ratio and LW, had microbiability estimates that were consistently significantly higher than 0 (Fig. 1) and (see Additional file 2: Table S2). These three traits all had RF microbiability estimates that were higher than the corresponding heritability estimate, which resulted in higher prediction accuracies of RF MV than BV for the same set of individuals. The two direct methane traits, CH4 and CH4Ratio, had RB microbiabilities that were lower than the corresponding heritability estimate, but higher prediction accuracies; while LW had RB microbiabilities that were lower than the corresponding heritability estimate and lower accuracy estimates. These results are consistent with the key role that the rumen microbiome plays in digestion of feed, of which methane is a by-product, and liveweight is a result of the energy made available to the animal by the rumen fermentation process.

The final methane-related trait, CO2, had microbiability estimates significantly higher than 0 only for phenotypes and rumen samples collected on lambs fed lucerne pellets (see Additional file 2: Table S2). While methane emissions are a by-product of ruminal bacterial digestion of food that the individual eats, carbon dioxide emissions are largely related to the basal metabolism of the animal, with a small portion produced by the rumen microbes during fermentation. Therefore, in a typical grazing situation (lambs and adults grazing on pasture), carbon dioxide emissions are likely to be largely driven by host genetics, as observed by the moderate heritability estimates. In the lucerne lamb group, the individuals were part of a feed intake trial with a more controlled environment (feeding regime and diet) than sheep grazing on pasture, therefore the variation in rumen microbial function explained a greater proportion of the phenotypic variation and the microbiability estimate was significantly higher than 0 for all methane-related traits.

### Incorporating metagenome profiles: residual feed intake

Residual feed intake is a complex trait, combining eating behaviour, rumination, and how the individual allocates the energy provided to it to different biological functions. RFI was measured on lambs fed an ad lib lucerne pellet diet, with a rumen sample taken during the trial. When using lucerne lamb metagenome profiles, microbiability estimates were significantly different from 0 when fitting M alone or when fitting G + M (Table 6). Prediction accuracy was higher for the MV (~ 0.47) than for the BV (0.29), and highest when using BV_G+M_ + MV_G+M_ (0.49), although only slightly higher than when using MV. The microbiability estimates and prediction accuracies for RFI from samples taken at the same time as RFI was collected were consistent with studies of RFI [34] and feed conversion ratio [35] in pigs, showing that this is observed across diverse species.

Microbiability of RFI using metagenome profiles from grass lambs or grass adults was also significantly different from 0, but their estimates and prediction accuracy were lower than for lucerne lambs (Table 6). When predicting RFI from a grass metagenome profile in addition to the BV, there was only a small increase in accuracy compared to using only the BV, suggesting minimal benefit from using rumen metagenome profiles on grass to improve predictions of RFI on a lucerne diet. This is likely due to the major role that the diet plays in rumen metagenome profiles [14, 36] as well as the differences in digestion of a grass diet vs. a lucerne pellet diet. Jonker et al. [18] showed that animals ranked similarly for methane emissions on a pellet vs. a pasture diet; however this may not be the case for RFI and this aspect needs further research.

### Incorporating metagenome profiles: prediction of methane-related traits and residual feed intake across age

For use as a practical predictive tool for selection of superior animals, it is critical that metagenome profiles are not only predictive of traits measured at the same time as the rumen sample is taken, but also across the lifetime of the animal. Ideally, a rumen sample taken early in life would be predictive of a range of environmentally and economically important traits across the individual’s life, including different environments they may experience e.g., different diets. Our dataset, with samples taken on the same individuals at different times in their life, is a great resource to begin exploring this relationship. We estimated microbiabilities and prediction accuracies for methane-related traits from lambs on grass using grass adult metagenome profiles, and microbiabilities and prediction accuracies from adults on grass using grass lamb metagenome profiles. These results showed that metagenome profiles can improve prediction accuracy beyond the models that just fit genetics, but as expected due to changes in the metagenome profile [14], the improvement was not quite as large as when samples were taken at the same time point as the phenotypes (Tables 5, 6). The prediction accuracy tended to be higher when using lamb metagenomes to predict adult phenotypes than vice versa – a favourable direction for being able to predict later life traits from an earlier rumen sample. Models predicting across age that fitted both animal genetic and metagenome effects simultaneously achieved similar prediction accuracies to the models that fitted only the metagenome effect when predicting using the metagenome profile from the same time point (Tables 5, 6). Therefore, we have shown that there is value in collecting metagenome samples early in life to predict performance of the individual later in life, particularly when an individual is expected to remain on a similar diet throughout its lifetime (e.g., ryegrass-based pasture).

### Incorporating metagenome profiles: health and production traits

The potential to improve prediction accuracy of health and production traits by incorporating metagenome profiles is attractive due to the direct economic impact that could be achieved. Previous studies have found mixed results for production traits such as sheep milk composition, with Martinez Boggio et al. [15] finding poor links between 16S rRNA sequencing operational taxonomic unit (OTU) and milk protein and fatty acid composition, while Bilton et al. [37] found significant microbiability estimates for milk fatty acid composition using RE-RRS. The three traits in this study, LW8, FEC1 and FW12, were selected to represent both production (LW8 and FW12) and health (FEC1) traits. Our results for LW8 (Table 7) and FW12 (see Additional file 2: Table S2) showed a slight increase in prediction accuracy from incorporating metagenome profiles collected at any diet/age evaluated when fitting a G + M model compared to just fitting G; however, the MV accuracies were lower than the BV accuracies. Conversely, results for FEC1 showed no improvement in prediction accuracy from fitting a model with metagenome profiles from grass lambs and microbiability estimates for lucerne lambs were equal to 0.

Incorporation of grass adult metagenome profiles did improve prediction accuracy of FEC1 compared to just fitting G (see Additional file 3: Table S3). Some of the tags included in the RF metagenome profiles were assigned to nematodes [14], and could potentially be indicative of current (or persistent) larval challenge, although the majority of economically important gastrointestinal nematode species in New Zealand sheep [38] are not represented in the databases used in that study. A more likely explanation for the improvement in prediction accuracy of FEC1 using metagenome profiles from grass adults is that adult rumen metagenome profiles may have been impacted by worm burdens that the individual experienced as a lamb, through the potential impact of treatment for nematode infection (e.g., anthelmintics) on the rumen microbiome [39], or changes in growth or metabolism as a result of nematode infection. Further work is required to explore these interactions; however, predicting FEC1 from grass adult metagenome profiles has no practical use, given that FEC1 is measured as a lamb.

### Metagenome modelling

Our study explored a variety of different approaches to generate an MRM for incorporation into a linear mixed model. We focused on two methods of generating metagenome profiles: (1) a reference-based approach which compared sequences to a set of high-quality bacterial and archaeal genomes from the Hungate1000 Collection [26]; and (2) a reference-free approach which counted the abundance of a set of common 65-bp sequences that were present in at least 25% of rumen samples from animals that were fed the same diet and were the same age. We also focused on two methods for normalizing the metagenome profiles, (1) L10: normalizing each genus (RB) or tag (RF) across all samples within the group; and (2) CA: normalizing each genus (RB) or tag (RF) within cohort.

#### Reference based vs. reference free

The RB microbiability estimates and accuracies all tended to be lower than the RF microbiability estimates, which is consistent with the increased proportion of reads that is captured using the RF approach compared to the RB approach. Hess et al. [14] reported that the RB approach captures ~ 20% of reads, while the RF approach captures ~ 30% when considering tags generated across all ~ 4500 samples (this percentage will be higher when considering tags present at least 25% within a group, as was done here). These results also support the findings of Hess et al. [17] on a smaller dataset, which showed much higher methane yield microbiability estimates for the RF than RB MRM. The RB approach captures a similar level of information on the metagenome profile as 16S rRNA gene sequencing as evidenced by similar estimates of CH_4_ microbiability of Holstein dairy cows found by Difford et al. [11] and Zhang et al. [40], which ranged from 0.07 to 0.15, depending on the modelling approach used, compared to our RB estimates for CH4 of 0.04 to 0.14. Hess et al. [17] showed that the MRM generated using the RB approach gave a greater microbiability estimate than the MRM generated from 16S rRNA gene sequencing data on the same samples. Our results suggest that the RF approach using RE-RRS may capture more of the phenotypic variation due to the rumen metagenome than 16S rRNA gene sequencing. The RF approach to metagenome profiling performs well using RE-RRS using the *Pst*I enzyme but may not perform as well for other sequencing approaches such as whole metagenome sequencing unless this is done at a (costly) very great depth. This was outlined by Hess et al. [17], and shown by RE-RRS with *Ape*KI, which captures a greater proportion of each genome (i.e., more tags) at less depth than *Pst*I, and gave a lower microbiability estimate than *Pst*I.

The reference database used for the RB approach will have a large impact on the relative performance of the RB and RF approaches. In our study, our reference database was based on the Hungate1000 Collection [26], augmented with four *Quinella* genomes [27]. This database contained very high-quality genome assemblies, with very high confidence in their taxonomic assignments. There are multiple ways that this database could be further expanded, including with genomes from a rumen MAG database (e.g., Stewart et al., [41] or Anderson and Fernando [42]), genomes from a more comprehensive database (e.g., GenBank [43]), or information from reference free tags [14]. Any additions to the reference database need to be carefully considered as they will increase computation time and have the potential to reduce accuracy if the genome assembly or taxonomic classification of the genome is incorrect; for example, GenBank taxonomies have been shown to have relatively high levels of incorrect taxonomic classification [44]. Reference database design and the performance of the RB and RF profiling approaches across a range of diets, ages and countries are discussed in more detail in Hess et al. [14].

The RF profiles contain information beyond the RB profiles, as they are able to capture a much broader taxonomic range, as shown in Hess et al. [14] and in Additional file 4: Table S4, which shows that RF tags can be assigned to bacteria, archaea, fungi, host and feed genomes, among others. However, many of the tags cannot be assigned, even with new and comprehensive databases. Additional file 4: Table S4 shows a relatively low proportion of reads assigned to the host (*Ovis aries*) genome, with only 0.29–0.48% of the RF profile attributed to the host, and even less, 0.15–0.17% attributed to plants. The majority of reads in the RF profiles can be attributed to bacteria, at 40–44% of assigned reads. The broader taxonomic range captured by the RF profiles has evidently resulted in an increase in prediction accuracy within and across age, flock, diet, and year. This indicates that the RF profiles are capturing valuable information that can be used for robust predictions. However, if the intention of an analysis with the RF approach is to focus on predictions using, e.g., only microbial data, the RF tags will need to have their taxonomy assigned and appropriate filtering applied. If the RF profiles are filtered in this way, they are limited by the accuracy and completeness of the reference databases used to perform the taxonomic assignment, similar to the RB approach as described above. This would also entail the removal of a large proportion of the RF profiles (using the GenBank database, 54–57% of the RF profile was still unassigned), and, most likely, a decrease in prediction accuracy. Given that grazing preference and host genetics are both heritable and repeatable, host genetics and feed composition captured by the RF profiles are valuable for prediction purposes, as outlined in this study.

#### Log-normalized vs. cohort-adjusted

The L10 and CA matrices gave very similar results for using both the RB and RF metagenome profiles, but the RFL10 model ran into convergence or singularity issues in several cases. This typically happened when the residual variance was zero, when the metagenome effect (or in the case of the G + M model, the animal genetic effect plus the metagenome effect) explained all the phenotypic variance. The RFCA model was selected for further analyses over the RFL10 model because there were fewer convergence or singularity issues observed. If there were fewer convergence or singularity issues, the RFL10 MRM may be more appealing than the RFCA MRM given the reduction in computation effort from normalizing each tag (L10) compared to normalizing each tag within cohort (CA). Care would need to be taken, however, when using a RFL10 MRM to predict across environments, e.g., different diets or even different flocks, because cohort was found to be a major driver of the observed relationships between samples by Hess et al. [14].

#### Convergence issues

The models fitting RF MRM, had convergence issues in some cases, most commonly when estimating prediction accuracy rather than heritability and repeatability (i.e., in the smaller cross-fold validation datasets rather than the full datasets). This generally happened when fitting both the GRM and MRM, and when these explained almost all the variation in the trait. In these cases, the model usually converged but the parameters did not, and continuing or rerunning ASReml did not achieve convergence. Given that the rumen microbiome/metagenome has been shown to be heritable [12–15], both matrices are able to capture genomic relationships between individuals, and therefore some of the variation in the trait could be assigned to either the GRM or MRM. As datasets get larger, models that capture the interaction between the GRM and MRM may be possible, however, these models were not possible with the dataset in our study. Although the model did not converge in all cases, the results (e.g., prediction accuracies) were still consistent with the models that considered only the GRM or only the MRM for that same trait, suggesting that the results were still reasonable, although the criteria for convergence were not reached. The increased frequency of convergence issues during cross-fold variation rather than with models using the full dataset highlights the importance of large datasets when performing analyses that integrate the metagenome and host genome.

#### Potential overfitting

Some of the models for CH4 had all or almost all of their variance explained by the combination of G and M when using the RF MRM. We expect that the comprehensive RF approach is likely to explain a very large portion of the variation in methane emissions because methane is produced only as a byproduct of bacterial fermentation of the feed in the rumen, i.e., there is no other source beyond random noise, either within the individual or its environment, that would contribute to the CH4 phenotype collected. This is particularly true when using the rumen sample collected at the same time as the phenotype was collected, and, supporting this, we see a drop in the variance explained by the combination of G and M when we use a metagenome profile collected at a different age. Considering CH4Ratio, compared to CH4, a decrease in the variation explained by the combination of G and M is observed, which is expected, given that CH4Ratio is influenced by both CH_4_ (driven by the rumen microbiome) and CO_2_, which is largely driven by other biological processes. RFI is another trait that tends to have a very high proportion of the variance explained by the combination of G and M. The rumen microbiome is known to have an impact on RFI, as the composition of microbes will impact how the feed is broken down into fatty acids to fuel the animal. Therefore, it makes sense that a large proportion of the variation in RFI can be attributed to the rumen metagenome. RFI is also under host genetic control, which captures the animal’s ability to use the available fatty acids, resulting in a very large proportion of the variation in RFI being explained by the combination of G and M. Although we expect the combination of the genome and the rumen metagenome to explain a large proportion of the variation in these traits, it was unexpected that we explained almost all the variation, so it is likely that we have an overfitting problem.

The RF models are very information-rich, with hundreds of thousands of tags represented, and the first principal components of these metagenome profiles explain only a small proportion of the variance of the matrices (e.g., less than 6.5% for *Pst*I; Hess et al. [17]). The difference in microbiability estimates between the RB and RF MRM is much larger than might be expected when considering the difference in prediction accuracies, which are still higher from the models using an RF MRM than those using an RB MRM. Some of the tags in the RF metagenome profile may be contributing to overfitting due to the increased dimensionality and generally lower average counts per column than the RB MRM. This is an example of the p >> n problem, where there are many more tags than individuals in the RF approach, compared to fewer genera than individuals in the RB approach. We ameliorated this issue by using an MRM to account for similarities in metagenome profiles between individuals rather than regressing on the individual tag abundance. The increase in prediction accuracy using the RF rather than the RB approach, indicates that the RF approach is picking up more meaningful associations than the RB approach, even if it is overfitting. This is likely driven by the broader taxonomic range that the RF metagenome profiles capture compared to the RB microbial profiles [14]. Future studies using the RF RE-RRS approach for metagenome profiling may benefit from further filtering or clustering of the tags to increase confidence in relative abundances and reduce the subsequent overfitting without reducing prediction accuracy. Hess et al. [14] explored the assignment of taxonomy to tags by comparison with a much more comprehensive database than would be possible with the RB approach and clustering tags based on these assigned taxonomies is one option that could be explored.

Another potential explanation for moderate accuracies in spite of some very high model fits is an interaction between the metagenome and the environment. This could be considered similar to G × E interactions between genomics and the environment, however, in the case of the metagenome, there could be very different abundances between different cohorts [36], and different interactions between the taxa in terms of abundances (i.e., different taxa playing the same role in feed degradation) [45], as well as different effects of those taxa in different environments (i.e., similar to what we consider to be G × E interactions). These interactions will become clearer as datasets grow bigger and we have more power to delve into the biological signals behind the predictions.

#### Metagenome profiles for selection purposes

When modelling metagenome profiles for the purposes of selection, it is important to consider the proportion of the phenotypic variance that is captured by the animal genetic effect (G) compared to the metagenome effect (M). There is extensive evidence that the abundance of certain rumen microbes, and therefore the microbiome/metagenome profile, is impacted by host genetics [12, 14, 40], indicating that selecting individuals with favourable metagenome profiles as parents for the next generation will result in offspring with favourable metagenome profiles and better performance. In our study, the models that we have run focused on phenotypic prediction, i.e., prediction of an individual’s performance, rather than that of their offspring. Further research is needed to confirm that the increase in prediction accuracy from fitting a model that incorporates rumen metagenome information will result in greater accuracy for predicting offspring performance. The impact of fitting both animal genetic and metagenome effects in the same model generally resulted in a decrease in both heritability (G_G+M_) and microbiability (M_G+M_) estimates compared to models fitting each independently (G_G_ and M_M_). This is because some of the phenotypic variation is being attributed to both the animal genetic and the metagenome effects when they are each fitted in separate models and is likely representative of the heritable variation in the microbiome that is influencing the trait of interest. Appropriate modelling of this variation is crucial to the development of models that will optimise our ability to identify individuals that have favourable genetics and microbiomes and will pass both to their offspring, e.g., identifying the heritable and non-heritable variation attributed to the metagenome [46–48].

## Conclusions

Metagenome profiles from rumen samples improved the accuracy of most animal traits evaluated in our study compared to models that considered only animal genomic relationships. The traits that we studied span environmentally important traits (methane emissions, methane ratio), efficiency traits (residual feed intake), production traits (liveweight at 8 months old, fleece weight at 12 months old) and health traits (summer strongyle faecal egg count). We recommend using a reference-free approach to metagenome profiling, rather than relying on a limited database of microbial genomes. The use of a cohort-adjusted reference-free microbial relationship matrix not only improved prediction accuracy of phenotypes recorded at the same time that the rumen sample was collected, but was also able to improve predictions at different ages and on different diets. Our study demonstrates the potential of using RE-RRS as a low-cost, high-throughput approach for generating metagenome profiles on thousands of animals for improved prediction of economically and environmentally important traits measured throughout an animal’s life.

## Supplementary Information

**Additional file 1: Table S1a**. Rumen sample information including SRA_number, sample ID, animal ID, flock, year of birth, sample date, diet, age, group, and cohort. **Table S1b**. Summary of Additional file 1 Table S1a, showing the number of samples per animal, overall and split into grass lamb, grass adult and lucerne lamb samples. **Table S1c**. Summary of Additional file 1: Table S1a, showing the number of animals in each cohort and which group the cohort belongs to

**Additional file 2: Table S2**. Variance components and prediction accuracies for methane-related traits including variance component, accuracy and regression coefficient estimates for the traits related to methane emissions (CH4), methane ratio (CH4Ratio), carbon dioxide emissions (CO2) and liveweight at the time of methane phenotype collection (LW).

**Additional file 3: Table S3**. Variance components and prediction accuracies for selected health and production traits including variance component, accuracy and regression coefficient estimates for the traits of liveweight at 8 months of age (LW8), summer strongyle faecal egg count (FEC1) and fleece weight at 12 months of age (FW12).

**Additional file 4: Table S4**: Proportion of RF profiles (reads) and number of tags attributed to various taxonomies of interest separated by group.

## Data Availability

The sequencing data analysed during the current study was generated as part of a study reported by Hess et al. [14] and is available in the NCBI Short Read Archive (SRA) database under BioProject ID PRJNA859547. The methane-related and residual feed intake data analysed during the current study are available from Suzanne Rowe on reasonable request. The health and production data analysed during this study are available from Sheep Improvement Limited or AgResearch, but restrictions apply to the availability of these data, which were used under license for the current study, and so are not publicly available.
